# The Ability to Form Homodimers Is Essential for RDM1 to Function in RNA-Directed DNA Methylation

**DOI:** 10.1371/journal.pone.0088190

**Published:** 2014-02-03

**Authors:** Taku Sasaki, Zdravko J. Lorković, Shih-Chieh Liang, Antonius J. M. Matzke, Marjori Matzke

**Affiliations:** 1 Institute of Plant and Microbial Biology, Academia Sinica, Taipei, Taiwan; 2 Gregor Mendel Institute of Molecular Plant Biology, Austrian Academy of Sciences, Vienna, Austria; University of Leeds, United Kingdom

## Abstract

RDM1 (RNA-DIRECTED DNA METHYLATION1) is a small plant-specific protein required for RNA-directed DNA methylation (RdDM). RDM1 interacts with RNA polymerase II (Pol II), ARGONAUTE4 (AGO4), and the *de novo* DNA methyltransferase DOMAINS REARRANGED METHYLTRANSFERASE2 (DRM2) and binds to methylated single stranded DNA. As the only protein identified so far that interacts directly with DRM2, RDM1 plays a pivotal role in the RdDM mechanism by linking the *de novo* DNA methyltransferase activity to AGO4, which binds short interfering RNAs (siRNAs) that presumably base-pair with Pol II or Pol V scaffold transcripts synthesized at target loci. RDM1 also acts together with the chromatin remodeler DEFECTIVE IN RNA-DIRECTED DNA METHYLATION1 (DRD1) and the structural-maintenance-of-chromosomes solo hinge protein DEFECTIVE IN MERISTEM SILENCING3 (DMS3) to form the DDR complex, which facilitates synthesis of Pol V scaffold transcripts. The manner in which RDM1 acts in both the DDR complex and as a factor bridging DRM2 and AGO4 remains unclear. RDM1 contains no known protein domains but a prior structural analysis suggested distinct regions that create a hydrophobic pocket and promote homodimer formation, respectively. We have tested several mutated forms of RDM1 altered in the predicted pocket and dimerization regions for their ability to complement defects in RdDM and transcriptional gene silencing, support synthesis of Pol V transcripts, form homodimers, and interact with DMS3. Our results indicate that the ability to form homodimers is essential for RDM1 to function fully in the RdDM pathway and may be particularly important during the *de novo* methylation step.

## Introduction

RNA-directed DNA methylation (RdDM) is a major pathway of short interfering RNA (siRNA)-guided epigenetic modifications in plants. RdDM is typified by methylation of cytosines in all sequence contexts (CG, CHG, CHH, where H is A, T or C) within the region of siRNA-DNA sequence homology. RdDM targets primarily transposons and other types of repeat, contributing to their transcriptional silencing and the maintenance of genome stability [1]–[3]. Genes containing transposon remnants in their promoter regions can also be targets of RdDM, which is implicated in a growing number of processes including pathogen defense [4]–[7], abiotic stress responses [8], [9], and gametophyte and embryonic development [10]–[12].

An intricate transcriptional machinery centered on two functionally-diversified, RNA polymerase II (Pol II)-related RNA polymerases, called Pol IV and Pol V, has evolved in plants and is specialized for RdDM [13]. Pol IV is required for producing the siRNA trigger for methylation whereas Pol V acts downstream to facilitate *de novo* methylation of DNA at the siRNA targeted site. Together with several accessory proteins, Pol V synthesizes scaffold transcripts that are thought to base-pair to siRNAs bound to ARGONAUTE4-clade proteins (AGO4/6/9), resulting in recruitment of DOMAINS REARRANGED METHYLTRANFERASE2 (DRM2) to catalyze *de novo* methylation at the DNA target site [14]. At some intergenic low-copy-number loci that do not ordinarily produce siRNAs, Pol II synthesizes scaffold transcripts that can similarly recruit AGO4/siRNAs to elicit transcriptional gene silencing (TGS). At other loci, Pol II transcription or transcripts can recruit Pol IV or Pol V to carry out their established roles in siRNA biogenesis and *de novo* methylation, respectively [15], [16].

One of the most enigmatic accessory components of the Pol V pathway is RNA-DIRECTED DNA METHYLATION1/DEFECTIVE IN MERISTEM SILENCING 7 (referred to hereafter as RDM1), a small, plant-specific protein of 163 amino acids [17]. RDM1 has a conserved DUF1950 domain but contains no other recognizable protein domains. Analysis of the crystal structure revealed that RDM1 contains a new protein fold that is unique to plants [18]. The crystal structure also demonstrated that the amino-terminal and carboxy-terminal parts of monomeric RDM1 are juxtaposed to create a hydrophobic pocket that binds a molecule of the hydrophobic detergent CHAPS. Gel filtration suggested that monomeric RDM1 forms a homodimer, which is supported by the crystal structure findings [18].

Both genetic and biochemical approaches have uncovered a role for RDM1 in RdDM and suggested various modes of action. RDM1 was retrieved in two independent forward genetic screens designed to identify mutants defective in RdDM and TGS [17]. Further analysis revealed that RDM1 is required for *de novo* methylation and that it interacts and co-localizes with Pol II, AGO4 and DRM2 in the nucleoplasm and Pol V in the perinucleolar processing center. RDM1 was reported to bind preferentially to single stranded DNA that is methylated in CHH nucleotide groups. This binding was weakened by a change in the hydrophobic pocket region of methionine-50 to alanine (M50A). The M50A mutation also rendered RDM1 nonfunctional in CHH methylation of several transposons and in reactivation of a silenced reporter gene in a *ros1* (REPRESSOR OF SILENCING1) mutant background [17]. These findings suggested that RDM1 plays a key role in targeting RdDM to specific sequences by linking DRM2 and AGO4, thus bringing the DNA methyltransferase activity to the siRNA-complementary site of the genome [17].

In another study, RDM1 was identified as a protein co-purifying with affinity-purified DEFECTIVE IN RNA-DIRECTED DNA METHYLATION (DRD1) and DEFECTIVE IN MERISTEM SILENCING3 (DMS3). DRD1 is a putative chromatin remodeler and DMS3 is a structural-maintenance-of-chromosomes hinge domain-containing protein. Both of these proteins were retrieved in forward genetic screens that identified Pol V pathway components [19] and have been shown to be necessary for Pol V recruitment to chromatin [20], [21]. Based on these data and their physical interactions, DRD1, DMS3 and RDM1 were proposed to form the DDR complex, which may facilitate synthesis of Pol V transcripts by remodeling chromatin ahead of Pol V [22]. How the proposed functions of RDM1 in the DDR complex and as a factor bridging DRM2 and AGO4 can be reconciled is still unclear.

To understand better the mode of action of RDM1 in RdDM, we generated altered versions that contain mutations in either the predicted dimerization or pocket regions and tested their performance in TGS, RdDM, synthesis of Pol V transcripts and protein interaction assays. As reported here, the results of our studies indicate that the ability to form homodimers is essential for RDM1 to function fully in RdDM and may be particularly crucial during the establishment of methylation.

## Results

**Figure 1:**
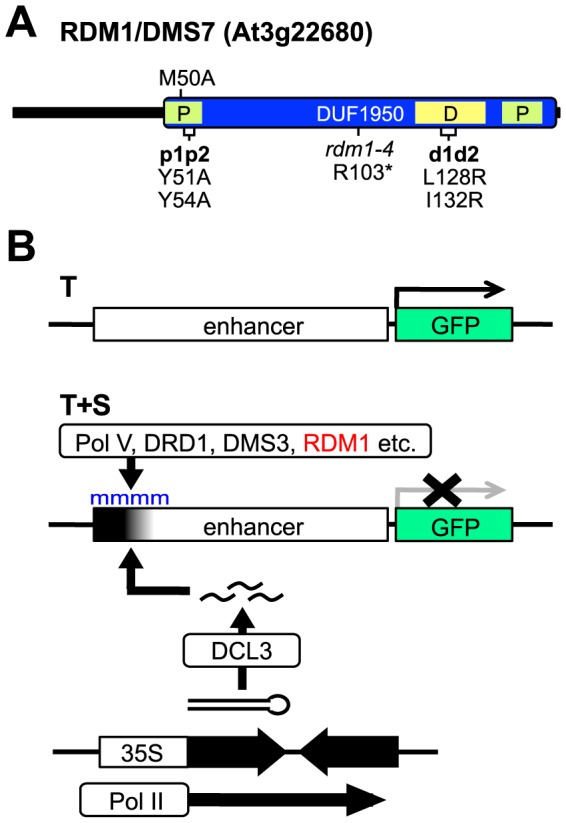
RDM1 structure and transgene-based meristem silencing system. A. RDM1 is 163 amino acids in length. The only recognizable protein domain is DUF1950. The *rdm1-4* mutation consists of a premature stop codon at arginine 103 [17]. The p1p2 (Y51A and Y54A) and M50A mutations are in the N-terminal part of the hydrophobic pocket domain (P). The d1d2 mutations (L128R and I132R) are in the putative dimerization region (D). B. The T+S transgene silencing system consists of a target locus (T) containing a *GFP* reporter gene under the control of an upstream enhancer, and a silencer locus (S), which contains a transcribed inverted DNA repeat (IR, opposing black arrows) of distal enhancer sequences (black shade). The IR is transcribed by Pol II from the 35S promoter, producing a hairpin RNA that is processed by DCL3 into 24-nt siRNAs, which trigger methylation (blue ‘m’) of the distal enhancer region and transcriptional silencing of the *GFP* gene in T+S plants. Methylation is dependent on Pol V transcription, which is proposed to be facilitated by the DDR complex (DRD1, DMS3, RDM1).

The *rdm1-4* allele, which contains a premature stop codon (R103*) (Fig. 1A), was isolated in our laboratory in a forward genetic screen for mutants defective in RdDM and TGS of a *GFP* reporter gene in *Arabidopsis thaliana* [17]. The screen was based on the T+S silencing system which comprises a target (T) locus encoding green fluorescent protein (GFP) and an unlinked silencer locus (S) encoding siRNAs targeted to an enhancer driving *GFP* expression (Fig. 1B). Wild-type T+S seedlings are GFP-negative owing to siRNA-induced methylation of the target enhancer [23]. *GFP* silencing is released and methylation of the target enhancer is lost in mutants defective in components of the Pol V pathway, including RDM1 [19].

To test the importance of the pocket and dimerization regions for RDM1 function in RdDM, we created three mutated versions of the *RDM1* gene. One version was designed to disrupt the putative hydrophobic pocket domain (mutations Y51A and Y54A; mutant termed *rdm1-p1p2*) and a second was designed to disrupt the putative dimerization domain (mutations L128R and I132R; mutant termed *rdm1-d1d2*) (Fig. 1A). In the third mutated version of RDM1, methionine-50 was changed to alanine (M50A). The M50 residue is also in the pocket region (Fig. 1A) and was previously reported to be essential for RDM1 to be fully functional in RdDM [17].

**Figure 2:**
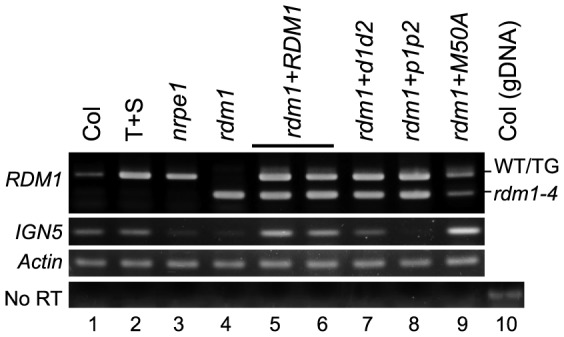
Mutated *rdm1* sequences are transcribed and differentially complement defects in Pol V transcription in an *rdm1* mutant. Top: Confirmation of *rdm1* transgene expression using RT-PCR. RNA from the corresponding genotypes was reverse transcribed into cDNA and amplified by PCR using gene-specific primers (Methods). The PCR product was then digested with *Pag*I (recognition site created by *rdm1-4* mutation). After *Pag*1 digestion, the transcript from the *rdm1-4* allele yields a shorter fragment around 250 bp (*rdm1-4*, lanes 4-9). The transcripts from a wild-type *RDM1* gene (lanes 1–3) or *rdm1-4* mutants complemented with the wild-type *RDM1* sequence (lane 5) or various mutated *rdm1* sequences (lanes 5–9) that lack the *rdm1-4* mutation remain undigested and produce a product around 500 bp (WT/TG). The larger PCR products were sequenced to confirm the expected mutations in the *rdm1-d1d2*, *rdm1*-*p1p2* and *rdm1*-*M50A* transgenes. Middle: Pol V-dependent *IGN5* transcripts are detectable by RT-PCR in wild-type Col-0 and T+S plants (lanes 1 and 2) and are reduced in *nrpe1* and *rdm1* mutants (lanes 3 and 4). *IGN5* transcripts can be detected after complementing the *rdm1* mutant with the wild-type *RDM1* construct (lanes 5 and 6) and with *rdm1*-*M50A* (lane 9). Low levels of *IGN5* transcript were observed with the *rdm1*-*d1d2* construct in an *rdm1* background(lane 7) but the *rdm1*-*p1p2* construct did not support transcription of *IGN5* above the level seen in the *rdm1* mutant (lane 8). RT-PCR analysis using actin primers is shown as loading control. Contamination by genomic was checked by omitting the RT step (No RT). Genomic DNA from a wild-type non-transgenic plant (Col) was used as positive control for the PCR reaction (No RT, lane 10).

**Figure 3:**
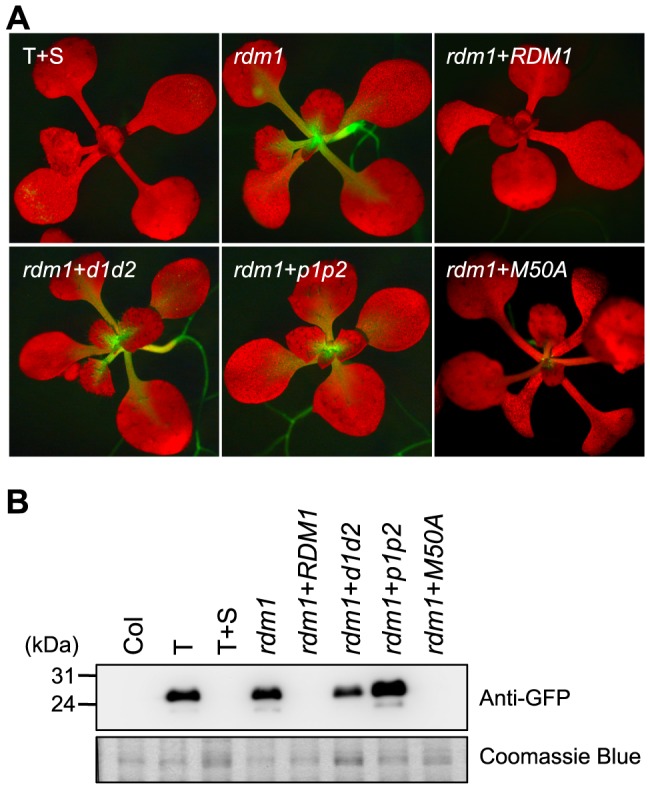
Mutated *rdm1* sequences differentially complement the *GFP* silencing defect in an *rdm1* mutant. *GFP* expression was detected by visualizing seedlings under a fluorescence microscope (A) and Western blotting using an antibody to GFP protein (B). *GFP* is active in T plants (part B only), silenced in wild-type T+S plants and released in the *rdm1* mutant. *GFP* silencing is restored when the mutation was complemented by wild-type *RMD1* genomic DNA (*rdm1+RDM1*). *GFP* silencing is not restored in an *rdm1* background after introducing constructs containing mutations in the putative dimerization domain (*rdm1+d1d2*) or pocket domain (*rdm1+p1p2*), whereas a construct containing the M50A mutation largely restored *GFP* silencing. In part B, Coomassie blue staining is shown as a loading control. Size markers in kD are shown at the left. The ‘Col’ lane shows non-transgenic control plants.

To assess the ability of the mutated versions of RDM1 to complement the deficiency in *GFP* silencing in the T+S system in an *rdm1* mutant, we introduced wild-type *RDM1* and the three mutated *rdm1* sequences under the control of the endogenous *RDM1* promoter into *rdm1* mutant plants. We used RT-PCR to confirm that the *rdm1* transgenes with the expected mutations were expressed in the *rdm1* mutant background in the respective transgenic lines (Fig. 2, *RDM1* panel). We then tested *GFP* expression by visualizing seedlings under a fluorescence microscope and by Western blotting using a GFP antibody (Fig. 3A and B, respectively). As reported previously [17], wild-type T+S seedlings are GFP-negative (Fig. 3, T+S) whereas the *rdm1-4* mutation substantially releases silencing to yield a GFP-positive phenotype (Fig. 3, *rdm1*). Introducing a wild-type *RDM1* sequence into the *rdm1* mutant restored *GFP* silencing (Fig. 3, *rdm1*+*RDM1*). Among the mutated versions of *RDM1*, only the *rdm1-M50A* sequence restored *GFP* silencing, although very weak *GFP* expression could be observed in the SAM in older seedlings (Fig. 3, *rdm1*+*M50A*). By contrast, *GFP* silencing was not restored after introducing either the *rdm1-p1p2* or *rdm1*- *d1d2* sequence into the *rdm1* mutant (Fig. 3, *rdm1*+*p1p2* and *rdm1*+*d1d2*).

**Figure 4:**
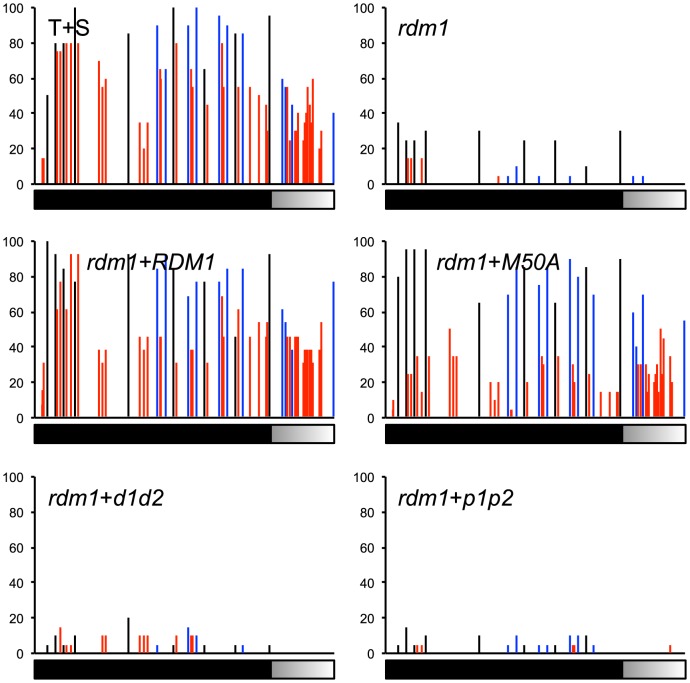
Mutated *rdm1* sequences differentially complement methylation defect at the target enhancer in an *rdm1* mutant. Bisulfite sequencing was used to assess DNA methylation at the target enhancer and downstream region (black and shaded bar) in the indicated genotypes. The Y-axis indicates the percent methylation at individual cytosines. The enhancer is methylated in wild-type T+S plants (CG, black; CHG, blue; CHH, red). This methylation is substantially reduced in the *rdm1* mutant but restored to wild-type levels after introducing the wild-type *RDM1* construct. Methylation is largely restored by the *rdm1-M50A* construct but not the *rdm1*-*d1d2* and *rdm1-p1p2* constructs.

We next examined methylation at the target enhancer upstream of the *GFP* reporter gene. As shown previously [17], in wild-type T+S plants that contain a silenced *GFP* gene the enhancer is densely methylated at cytosines in all sequence contexts (Fig. 4, T+S). By contrast, in the *rdm1* mutant, in which *GFP* silencing is released, this methylation is almost completely lost (Fig. 4, *rdm1*). The wild-type *RDM1* sequence fully restored methylation to the target enhancer in an *rdm1* mutant background (Fig. 4, *rdm1*+*RDM1*). Similarly, the *rdm1-M50A* sequence largely complemented the methylation deficit, with only a partial reduction of CHH methylation relative to wild-type the level (Fig. 4, *rdm1*+*M50A*). By contrast, neither the *rdm1-d1d2* nor *rdm1-p1p2* sequence was able to restore methylation of the target enhancer in an *rdm1* mutant background (Fig. 4, *rdm1*+*d1d2* and *rdm1*+*p1p2*).

**Figure 5:**
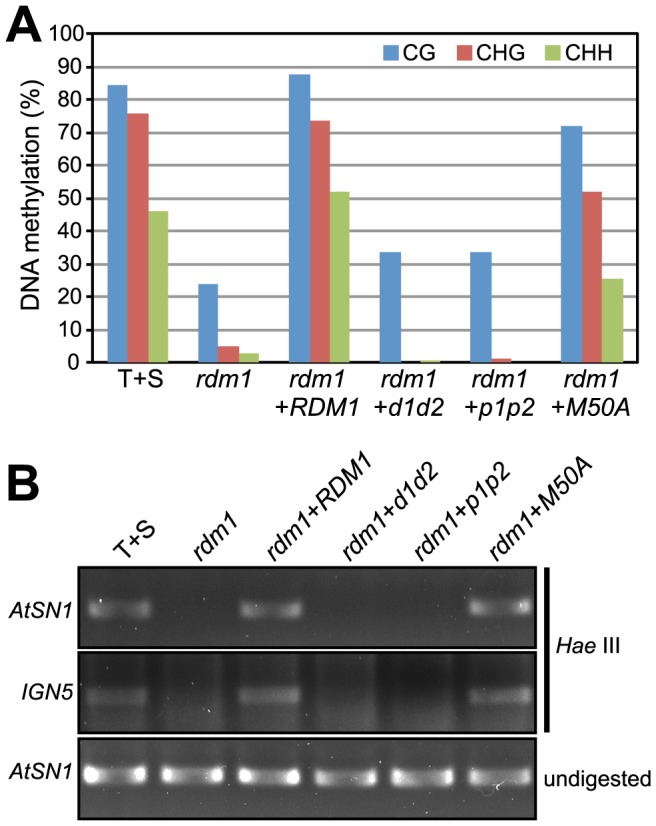
Mutated *rdm1* sequences differentially complement methylation defect at endogenous RdDM targets in an *rdm1* mutant. A. Bisulfite sequencing was used to assess DNA methylation at the endogenous *Tag2* element. The Y-axis indicates the percent methylation at individual cytosines. *Tag2* is methylated in wild-type T+S plants (CG, black; CHG, blue; CHH, red). This methylation decreases substantially in the *rdm1* mutant but returns to wild-type levels after introducing the wild-type *RDM1* construct. Methylation is largely restored after introducing the *rdm1*-*M50A* sequence but not the *rdm1*-*d1d2* and *rdm1*-*p1p2* sequences. B. Chop-PCR analysis to assess methylation at endogenous *AtSN1* and *IGN5* loci. The indicated genomic DNAs were digested by *Hae* III, which is sensitive to CHH methylation, and used as templates in PCR amplification using primers flanking the restriction enzyme site. Detection of the amplified fragment indicates the presence of CHH methylation. Undigested *AtSN1* amplification products are shown as a loading control.

We made comparable observations at several endogenous loci. The *Tag2* locus loses substantial methylation in all sequence contexts in the *rdm1* mutant compared to wild-type plants (Fig. 5A, *rdm1* and T+S). Neither the *rdm1-p1p2* nor *rdm1-d1d2* sequence was able to complement the defect in DNA methylation (Fig. 5A, *rdm1*+*p1p2* and *rdm1*+*d1d2*). By contrast, the wild-type *RDM1* sequence fully restored and the *rdm1-M50A* sequence largely restored the wild-type levels of DNA methylation (Fig. 5A, *rdm1*+*RDM1* and *rdm1*+*M50A*). Similarly, at the *AtSN1* and the *IGN5* loci, neither *rdm1-p1p2* nor *rdm1-d1d2* restored methylation whereas *rdm1-M50A* and the wild-type *RDM1* sequence were able to re-establish methylation (Fig. 5B).

We used RT-PCR to examine synthesis of the Pol V intergenic transcript *IGN5* in the *rdm1* mutant complemented with the three *rdm1* mutated sequences. As expected, the *IGN5* transcript was detectable in wild-type plants (Fig. 2, *IGN5* panel, lanes 1 and 2) but absent in the Pol V-defective mutant *nrpe1* and in *rdm1* (Fig. 2, lanes 3 and 4). After introducing the wild-type *RDM1* sequence into the *rdm1* mutant, *IGN5* transcripts were again detectable (Fig. 2, two independent lines, lanes 5 and 6). Similarly, *IGN5* transcripts were detected after introducing the *rdm1-M50A* sequence into *rdm1* (Fig. 2, lane 9). The *IGN5* transcript was also observed, but at a somewhat lower level, after introducing the *rdm1-d1d2* sequence (Fig. 2, lane 7). However, introduction of the *rdm1-p1p2* sequence into the *rdm1* mutant did not restore *IGN5* transcription (Fig. 2, lane 8).

**Figure 6:**
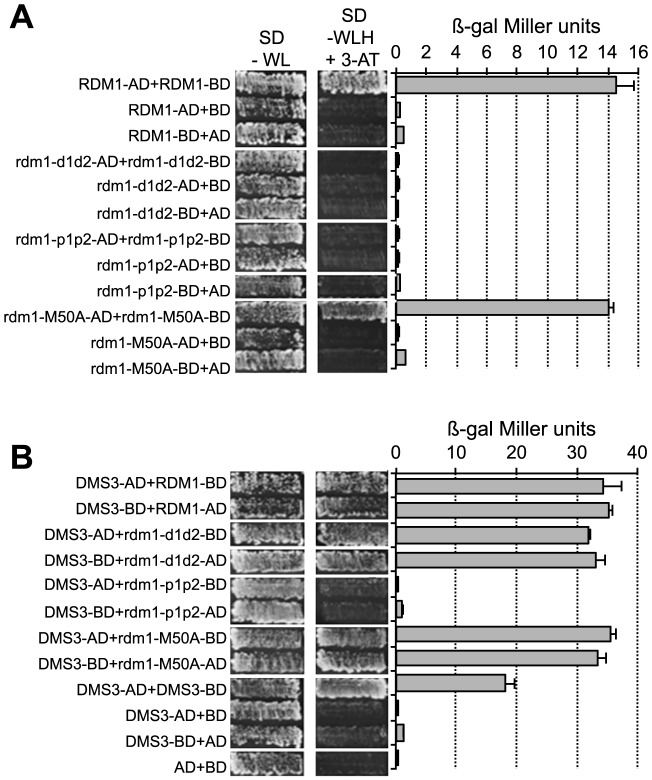
Yeast two-hybrid assays to test homodimerization and DMS3 interactions of mutated rdm1 proteins. **A**. Test for homodimer formation by wild-type RDM1 and three mutated versions of the protein. **B**. Test for interaction of wild-type and mutated forms of RDM1 with DMS3. In A and B the indicated combinations of plasmids were transformed into Hf7c yeast reporter strain and assayed for growth on synthetic medium (SD) plates without tryptophan and leucine (SD-WL) or without tryptophan, leucine and histidine (SD-WLH) supplemented with 10 mM 3-amino-1,2,4-triazole.

Using yeast two-hybrid assays, we tested wild-type RDM1 and the three mutated versions for their ability to form homodimers and to interact with DMS3. In accord with previous results [17], wild-type RDM1 formed homodimers in this assay as evidenced by activation of his3 (growth on synthetic medium without histidine – SD-WLH+3-AT) and β-galactosidase reporter genes (Fig. 6A, RDM1). However, of the three mutated versions, only rdm1-M50A was able to form homodimers, with β-galactosidase activity being comparable to that of wild-type protein (Fig. 6A, rdm1-M50A). Neither rdm1-d1d2 nor rdm1-p1p2 was able to form homodimers (Fig. 6A). Because none of the constructs activated reporter genes when co-transformed with the respective empty plasmids, we conclude that the observed interactions are specific.

Consistent with their association in the DDR complex [22], wild-type versions of RDM1 and DMS3 interacted strongly in a yeast two-hybrid assay (Fig. 6B, first two rows). The rdm1-d1d2 and rdm1-M50A variants also interacted with DMS3 at levels similar to wild-type RDM1 (Fig. 6B, DMS3+rdm1-d1d2 and DMS3-M50A). Of the three mutated versions, only rdm1-p1p2 failed to interact with DMS3 (Fig. 6B, DMS3+rdm1-p1p2). As suggested previously [23], DMS3 also forms homodimers in a yeast two-hybrid assay (Fig. 6B, DMS3).

## Discussion

RDM1 is a core component of the Pol V branch of the RdDM pathway [1]–[3] but its mode of action is still unclear. RDM1 is implicated in both the DDR complex [22], which facilitates Pol V transcription, and in linking DRM2 and AGO4 during the *de novo* methylation step when AGO4-bound siRNAs presumably interact with Pol II or Pol V-generated scaffold transcripts at target loci [17]. We tested wild-type and various mutated versions of RDM1 for their ability to complement defects in RdDM/TGS, to support synthesis of Pol V transcripts, and to form homodimers and interact with DMS3. Our results demonstrate that the ability to form homodimers is a fundamental requirement for RDM1 to be fully functional in the RdDM pathway. Our findings also suggest that RDM1 may act as a monomer and homodimer, respectively, during its proposed roles in the DDR complex and during the *de novo* methylation step, when it has been suggested to act as a bridging protein between AGO4 and DRM2.

**Figure 7:**
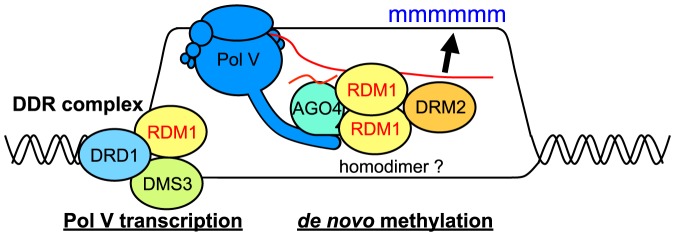
Model of differential RDM1 participation in the RdDM pathway. Our data are consistent with a hypothetical model in which RDM1 acts as a monomer in the DDR complex (DRD1, DMS3, RDM1), which facilitates Pol V transcription, and as a homodimer during the *de novo* methylation step when it has been proposed to act as a bridging protein between AGO4 and DRM2 [17]. AGO4 interacts with the C-terminal domain of the largest subunit of Pol V (NRPE1) and binds the siRNA guide (short red wavy line), which base-pairs with a nascent Pol V transcript (longer red line), thus targeting DRM2-catalyzed methylation (blue ‘m’) to the homologous DNA target site.

In agreement with previous results showing that affinity purified RDM1 and DMS3 co-purify with each other [22], we found that these two proteins interact strongly in a yeast-two hybrid assay. Both proteins are also capable of forming homodimers in this assay. As expected, the wild-type *RDM1* sequence fully complemented all deficiencies of the *rdm1* mutant in the tests performed. However, the mutated *rdm1* sequences differed in their abilities to complement defects resulting from the *rdm1* mutation (Table 1). Whereas *rdm1-M50A* restored wild-type or nearly wild-type function in all assays, the *rdm1-p1p2* sequence did not complement any of the *rdm1* mutant phenotypes. By contrast, mixed complementation results were obtained with *rdm1-d1d2*. Although able to interact with DMS3 and support synthesis of Pol V transcripts, indicating formation of a functional DDR complex, the rdm1-d1d2 protein was unable to form homodimers or to complement defects in RdDM and TGS. These findings suggest that the ability to form homodimers is dispensable for the action of RDM1 in the DDR complex but essential for its other proposed role in *de novo* methylation (Fig. 7). Determining whether RDM1 indeed interacts with AGO4 and DRM2 as a homodimer requires further biochemical work.

**Table 1:** Summary of results.

	GFP silencing (Figure 3)	RdDM (Figure 4, 5)	Pol V transcription (Figure 2)	Homodimer (Figure 6)	Interaction with DMS3 (Figure 6)
*rdm1+DMS7*	yes	yes	yes	yes	yes
*rdm1+d1d2*	no	no	weak	no	yes
*rdm1+p1p2*	no	no	no	no	no
*rdm1+M50A*	yes	CHH reduced	yes	yes	yes

The reason for the differential requirement for homodimerization of RDM1 during these two steps of the RdDM mechanism is not yet known. An RDM1 homodimer contains two hydrophobic pockets instead of one, which increases the number of binding sites for potential ligands, but the functional significance of this difference remains unclear. Because previous biochemical work suggested that the other DDR complex members, DRD1 and DMS3, may be present in multiple complexes [22], it is not inconceivable to suggest that RDM1 – as either a monomer or homodimer - also participates in more than one step or complex in the Pol V pathway. It is also possible that the ability to form homodimers in the yeast two-hybrid assay is unrelated to homodimer formation in vivo and rather reflects another important feature of the RDM1 protein that remains to be defined in more detail. Additional biochemical assays will be required to obtain insights into the structure, stability and dimeric status of the RDM1 and RDM1 mutants and their interactions with DMS3.

The inability of the rdm1-d1d2 protein, which contained two mutations in the predicted dimerization region, to form homodimers was anticipated. However, it remains unclear why the rdm1-p1p2 protein, which contains two mutations in the proposed hydrophobic pocket region, also failed to form homodimers. Because of this, we were unable to investigate the putative pocket mutations (p1p2) separately from mutations that affected homodimer formation. The further inability of the rdm1-p1p2 protein to interact with DMS3, in contrast to rdm1-d1d2, may suggest that the pocket region is important for interactions with DMS3. However, the single M50A change in the pocket region did not substantially disrupt interactions with DMS3. Perhaps multiple changes in hydrophobic residues, such as those present in the rdm1-p1p1 protein, are necessary to disrupt the proposed DMS3 binding function of the pocket region.

Our results showing that introduction of the *rdm1-M50A* construct into the *rdm1* mutant largely restores wild-type function in all assays performed contrast with previous results suggesting that the M50A mutation renders RDM1 nonfunctional in RdDM, gene silencing and binding to methylated DNA [17]. We did observe some reduction of CHH methylation at the transgene enhancer and the endogenous *Tag2* element as well as somewhat leaky restoration of *GFP* silencing in the SAM of older *rdm1-M50A* mutant seedlings, suggesting marginal reductions in RdDM efficiency. However, the levels of CG and CHG methylation in the *rdm1-M50A* mutant were only slightly lower than those observed following complementation with the wild-type *RDM1* sequence. We also observed essentially wild-type levels of CHH methylation at *AtSN1* and the *IGN5* locus in the *rdm1-M50A* mutant, whereas a previous report indicated substantial loss of CHH methylation of *AtSN1* in an *rdm1-M50A* mutant background [17]. Locus-specific effects are thus unlikely to account for the discrepant results. A more probable explanation is that the activity of the *rdm1-M50A* transgene was tested in different genetic backgrounds. In the study of Gao and coworkers, the complementation ability of *rdm1-M50A* was examined in a *ros1* mutant background, in which DNA methylation accumulates to higher levels than in the *ROS1* wild-type background used in this study, owing to defects in removing DNA methylation. It is also possible that the two different *rdm1-M50A* transgenic lines, which were produced independently in two different labs, differ (for unknown reasons) in the extent to which the M50A mutation affects the function of RDM1 in RdDM.

RDM1 has a crucial role in the RdDM mechanism and is arguably the most novel protein discovered so far in this pathway. Yet, the evolutionary origin of RDM1 is obscure. So far, clear RDM1 homologs are found only in flowering plants and there are no related proteins in other organisms. BLAST searches retrieve several larger RDM1-related proteins in legumes (*Medicago truncatula* MTR_5g006600, 369 amino acids and *Cicer arietinum* LOC101512373, 350 amino acids), which have unique N-terminal extensions of unknown function. Interestingly, a 171 amino acid protein in *M. truncatula* (MTR_5g006580) appears to be a fusion protein containing part of POLD4, the smallest subunit of DNA polymerase delta [24], and DUF1950, which is also found in RDM1. POLD4, which ranges in length between 100-120 amino acids in different plant species, has been proposed to stabilize the POLD-PCNA complex during lagging strand synthesis [24]. Although this fusion protein appears to be unique to *M. truncatula*, it nevertheless illustrates a putative functional connection between RDM1 and a small polymerase subunit. Additionally, RDM1 was found to show weak structural homology to a bacterial RNA polymerase ơ factor [18]. Although it is premature to draw conclusions from these observations, it is conceivable that RDM1 evolved from a small polymerase subunit or co-factor and eventually became specialized for the Pol V branch of the RdDM pathway.

## Materials and Methods

### Plant materials

All experiments reported here were performed with *Arabidopsis thaliana* ecotype Col-0. The isolation of *rdm1-4*/*dms7-1* was reported previously [17]. The *nrpe1-10* allele [23] was used in reverse transcription-polymerase chain reaction (RT-PCR) experiments to test for *IGN5* transcription. The wild-type and mutated versions of *RDM1* DNA (*rdm1*-*p1p2, rdm1-d1d2*, and *rdm1-M50A*) were generated by Mr. Gene GmbH (Regensburg), ligated into the MpPATot binary vector [25], and introduced into the *rdm1-4* mutant using the floral dip method [26]. Transformants were selected for resistance to phosphinothricin on solid Murashige and Skoog medium. All transgenic lines were produced in our laboratory in the same T+S *rdm1-4* mutant background.

### DNA methylation analysis

For bisulfite-sequencing analysis of DNA methylation, genomic DNA was isolated using DNeasy Plant Mini Kit (Qiagen). Bisulfite treatment using EpiTect Bisulfite Kit (Qiagen) and subsequent sequencing were performed as described previously [27]. For bisulfite sequencing, the following primers were used for the enhancer region of the target transgene: EPRV-Top2F (5′-GCG GTG TYA TYT ATG TTA YTA GAT-3′) and EPRV_Top2R (5′-CTT CTT RAT RTT CCA TAR CTT TCC-3′). For the endogenous Tag2 sequence, the primers used were: TAG2_top1 (5′-YTT AGT GGG AAG ATT YAG AAG TA-3′) and TAG2_top2 (5′-CAT RTC CAT RAR CAA CCC ATT RT-3′).

Chop-PCR was used to assess CHH methylation at the *AtSN1* and *IGN5* loci. Chop-PCR involves digesting genomic DNA with a methylation-sensitive restriction enzyme followed by PCR amplification using primers flanking the restriction enzyme site. For this, 50ng genomic DNA was digested with *Hae*III (which reports on CHH methylation) in a 20 µl reaction mix, and 1 µl of digested DNA was used as a template for PCR. Primers used for chop-PCR were as follows; for *AtSN1*, AtSN1for (5′-ACC AAC GTG CTG TTG GCC CAG TGG TAA ATC-3′) and AtSN1rev (5′-AAA ATA AGT GGT GGT TGT ACA AGC-3′), and for *IGN5*, IGN5for (5′-TCC CGA GAA GAG TAG AAC AAA TGC TAA AA-3′) and IGN5rev (5′-CTG AGG TAT TCC ATA GCC CCT GAT CC-3′).

### Plasmids

RDM1, RDM1d1d2 and DMS3 yeast two-hybrid plasmids have been described previously [17], [28]. *rdm1-p1p2* and *rdm1-M50A* mutated sequences were synthesized by Mr. Gene GmbH (Regensburg) and cloned into yeast two-hybrid plasmid in the same way as *rdm1* and *rdm1-d1d2* [17].

### Yeast two-hybrid assay

Transformation of the yeast reporter strain Hf7c, selection of transformants, test for activation of His3 reporter gene and measurements of β-galactosidase activities were carried out according to manufacturer's instructions (Clontech). For measuring β-galactosidase activity, two independent colonies were selected and from each culture β-galactosidase activity was determined in triplicates with ONPG as a substrate.

### Western blotting to detect GFP protein

Approximately 0.1 g of seedlings were ground in liquid nitrogen and proteins were extracted in extraction buffer (50 mM Tris –HCl, pH 7.5, 5 mM EDTA, 150 mM NaCl, 0.1% Triton X-100, 0.2% NP-40). Five micrograms of protein extracts were separated by sodium dodecylsulfate (SDS) polyacrylamide gel electrophoresis (PAGE) using 10% acrylamide gels. After SDS-PAGE, proteins were transferred onto a polyvinylidene difluoride (PVDF) membrane (Bio-Rad). The membrane was then incubated with blocking reagent (10% w/v skim milk in 0.02% Tween 20-TBS [TTBS]) for 1 hr at room temperature. GFP was targeted by incubating the membrane with the 1∶1000 diluted anti-GFP antibody (Roche) at 4°C for overnight. After the membrane was washed with 0.02% TTBS, the secondary antibody was added [1∶3000 anti-mouse IgG-HRP (Bio-Rad)] and incubated with the membrane for 1 hr at room temperature. The signal was detected by BioSpectrum Imaging System (UVP, Jena) after washing and applying enhanced chemiluminescence (ECL) substrates (Amersham).

### RT-PCR to detect RNA Pol V transcript IGN5 and rdm1 transgene expression

Total RNA from 2-week-old seedlings was extracted using an RNA isolation kit (Genemark). The Pol V transcript *IGN5* was detected using a previously published procedure [20]. In brief, 1 µg fresh RNA was reverse transcribed by 0.25 µM IGN5 gene specific primers (5′-TCC CGA GAA GAG TAG AAC AAA TGC TAA AA-3′ and 5′- CTG AGG TAT TCC ATA GCC CCT GAT CC-3′) and the resulting cDNA was amplified by using a One-Step RT-PCR kit (Invitrogen, Catalog number 10928-042). Reverse transcription was carried out at 55°C for 30 min and followed by 70°C for 15 min. After cDNA synthesis, 1 µl of 12.5 µM IGN5 primer mixture was added for the PCR reaction. The PCR reaction cycle included an initial denaturation step (94°C for 2 min), and 35 cycles of amplification (94°C for 30 sec, 60°C for 30 sec, 72°C for 45 sec). For a loading control on the gel, the same amount of RNA was reverse transcribed by using Transcriptor First Strand cDNA Synthesis Kit (Roche, Catalog number 04897030001). The resulting cDNA was diluted 10 times and then used as the template for following PCR analysis. As a constitutively expressed control, actin was amplified for 23 cycles by primers 5′-TCG TGG TGG TGA GTT TGT TAC-3′ and 5′-CAG CAT CAT CAC AAG CAT CC-3′. The thermo cycles of PCR were 98°C for 10 sec, 55°C for 30 sec, 72°C for 30 sec, with an extra final extension at 72°C for 5 minutes.

Transcription of the *rdm1* transgenes was confirmed by cleaved amplified polymorphic sequences (CAPS) analysis using cDNAs as templates. PCR products were amplified using as primers DMS7tgF2 (5′-CCG GTT CAT CTG ACG TCG ATG C-3′) and DMS7tgR2 (5′-AAG CTT CAA TGC TTG AAC AAG G-3′), and then digested with *Pag* I. The *rdm1-4* mutation creates a site for *Pag*1 and an approximately 250 bp transcript is detectable in the *rdm1-4* mutant following *Pag*1 digestion of the PCR product. The wild-type *RDM1* sequences and the *rdm1-p1p2*, *rdm1-d1d2* or *rdm1-M50A* sequences do not contain the *Pag*1 site and the transcript detected following *Pag*1 digestion of the PCR product is around 500 bp. The 500 bp fragments were sequenced to confirm the presence of the expected mutations in *rdm1-p1p2*, *rdm1-d1d2* and *rdm1-M50A* (Fig. 2, *RDM1*, lanes 7-9).
